# Commentary: Ketone Diester Ingestion Impairs Time-Trial Performance in Professional Cyclists

**DOI:** 10.3389/fphys.2018.00279

**Published:** 2018-03-27

**Authors:** Brianna J. Stubbs, Andrew P. Koutnik, Angela M. Poff, Kenneth M. Ford, Dominic P. D'Agostino

**Affiliations:** ^1^HVMN Inc., San Francisco, CA, United States; ^2^Department of Physiology, Anatomy and Genetics, University of Oxford, Oxford, United Kingdom; ^3^Molecular Pharmacology and Physiology, University of South Florida, Tampa, FL, United States; ^4^Florida Institute for Human and Machine Cognition, Pensacola, FL, United States

**Keywords:** ketones, ketone ester, ketone salt, beta-hydroxybutyrate, acetoacetate, cycling, time trial, performance

We read with interest the recent article by Leckey et al., reporting that consumption of a ketone diester drink impaired 31 km cycling time trial performance (Leckey et al., 2017). Exogenous ketones are new to the field of sports science, with only six athlete studies published to date (Cox et al., 2016; Holdsworth et al., 2017; O'Malley et al., 2017; Rodger et al., 2017; Vandoorne et al., 2017; Evans et al., 2018). Therefore, this article is a welcomed contribution to the discussion surrounding their optimal use. Having worked with novel ketone compounds in animals, sedentary individuals, and athletes, we wish to offer some reflections on this study, which may assist with future investigations of exogenous ketones in athletics.

We suggest that fully understanding the human pharmacokinetics of novel ketone compounds will permit optimal dosing. Reaching blood ketone concentrations >2 mM is likely a key mediator of any potential ergogenic effect (Cox et al., 2016; Egan and D'Agostino, 2016; Evans et al., 2017). Blood ketone kinetics after identical doses of a beta-hydroxybutyrate (BHB) monoester or acetoacetate (AcAc) diester are unlikely to be comparable with respect to BHB and AcAc. These esters deliver a different number of “ketone equivalents.” The BHB monoester delivers two BHB equivalents. The AcAc diester delivers two AcAc equivalents and one racemic BHB equivalent. Even if ketone equivalents were matched, different compounds have distinct pharmacokinetics. For example, ketone levels after the BHB monoester differed to those after matched quantities of racemic BHB salts (Stubbs et al., 2017). Therefore, future studies should ensure that the dose chosen delivers sufficient blood ketone concentrations.

There are metabolic differences between the ketone molecules delivered by exogenous ketones. This can impact their physiological and neurological effects. Optical isoforms of BHB are not equivalent: d-BHB is released by the liver, whereas l-BHB is an intracellular metabolite. It appears that l-BHB less readily undergoes oxidative metabolism (Webber and Edmond, 1977; Desrochers et al., 1992); therefore elevating l-BHB may not improve athletic performance. Furthermore, there may be different effects of BHB and AcAc. Elevating AcAc can oxidize the mitochondria compared to BHB. Some AcAc is reduced to BHB, generating NAD^+^ from NADH; this could alter muscle energy metabolism. BHB and AcAc possess distinct signaling properties, and thus different physiological effects on cerebral (D'Agostino et al., 2013) and tumor metabolism (Poff et al., 2014). These data highlight the importance of distinguishing between d-BHB, l-BHB, and AcAc, and accounting for this in study design and interpretation.

In our view, and as is mentioned throughout Leckey et al., a probable cause of performance impairment was the frequency and severity of GI distress experienced by athletes consuming the AcAc diester. Currently, the AcAc diester remains unpalatable, with limited GI tolerability. However, formulation and dosing of ketone esters can be refined to increase palatability. In early studies of the BHB monoester given in a milkshake, GI symptoms occurred when three drinks were taken daily for 5 days (2,142 mg/kg), but no symptoms were reported with a single bolus (Clarke et al., 2012). Tolerability was further improved using a flavored water, and in Cox et al. (2016) and Holdsworth et al. (2017), there were no severe symptoms that influenced exercise capacity. Given that similar levels of ketosis were reached using an IV infusion without GI effects (Mikkelsen et al., 2015), it seems unlikely that hyper-ketonemia is responsible for the symptoms reported by Leckey et al. While consumption of a fizzy beverage may mitigate poor flavoring, it would likely worsen symptoms. It is unadvisable that athletes consume any novel supplement for the first time in competition (even common substances such as caffeine and bicarbonate) in case ill-effects occur. Therefore, we suggest that before using ketone supplements in a performance setting, tolerability and dosing should be optimized. Removing the confounding influence of GI distress will help elucidate ketone-specific effects on performance.

Finally, we wish to highlight the importance of analytical techniques in the study of ketone metabolism. Enzymatic assays of BHB only detect the d-isoform, not l-BHB; the only way to accurately detect both is using mass spectrometry analysis. Additionally, AcAc is unstable and rapidly degrades when stored for over 48 h (Price et al., 1977). Hence, analysis should be undertaken immediately to avoid underestimation. Alternatively, treating AcAc samples with NaBH_4_ converts AcAc into a stabilized BHB derivative, which can be analyzed after longer storage (Lincoln et al., 1987; D'Agostino et al., 2013). The low levels of AcAc reported by Leckey et al., are inconsistent with previous work using the AcAc diester, suggesting degradation may have occurred. While urine strip testing for AcAc offers convenient, non-quantitative assessment of AcAc excretion, it does not correlate to blood ketone levels after exogenous ketones (Stubbs et al., 2017). If blood AcAc concentration cannot be measured, urine AcAc concentration should be quantified and total excretion calculated using urinary volume.

To conclude, consumption of the AcAc diester was ergolytic in a 31 km cycling time trial in the doses and formulation studied by Leckey et al. However, it is unclear if this would be the case were dosing and formulation optimized to achieve higher BHB levels and improve tolerability. Given the nature of the GI symptoms described, it is unknown whether hyper-ketonemia *per-se* impaired performance, as implied in the title and abstract of this paper. Contrasting results from existing studies using different ketone compounds, performance tests, dosages, and analytic techniques highlight that further research is required to determine the role of exogenous ketones in athletic performance (Cox et al., 2016; Leckey et al., 2017; O'Malley et al., 2017). Additionally, emerging research suggests that exogenous ketones could augment athlete recovery (Holdsworth et al., 2017; Vandoorne et al., 2017), and may be an effective training aid. Taken together, ketone supplements allow exploration of a novel physiological state, where ketones can be present on a background of replete carbohydrate reserves. We hope that the significant work undertaken by Leckey et al., along with the recommendations here will guide future research into the effects of ketones on performance and health.

## Author contributions

All authors agreed on discussion points for the commentary. BS wrote the initial draft with help from AK. AP, KF, and DD extensively revised and edited commentary. All authors reviewed and agreed final version prior to submission.

### Conflict of interest statement

BS is an employee of HVMN Inc., which sells exogenous ketone products. She has the option to purchase stock in HVMN Inc. AP is a scientific consultant for Pruvit Ventures, which sells exogenous ketone supplement products. She is also an inventor on intellectual property related to exogenous ketone supplementation for various uses. DD is an inventor on intellectual property related to exogenous ketone supplementation for various uses. The other authors declare that the research was conducted in the absence of any commercial or financial relationships that could be construed as a potential conflict of interest.

## Abbreviations

AcAc: acetoacetate
BHB: beta-hydroxybutyrate
GI: gastro intestinal.
